# Duration of reappearance of gingival melanin pigmentation after surgical removal — A clinical study

**DOI:** 10.4103/0972-124X.70828

**Published:** 2010

**Authors:** Harjit Kaur, Sanjeev Jain, Roshan Lal Sharma

**Affiliations:** *Department of Periodontology and Oral Implantology, Guru Nanak Dev Dental College and Research Institute, Sunam, India*; 1 *Department of Periodontology and Oral Implantology, Government Dental College and Hospital, Patiala, Punjab, India*

**Keywords:** De-epithelialization, gingival melanin pigmentation, repigmentation

## Abstract

**Background:**

In dentistry, esthetics has a special place. Although gingival melanin pigmentation does not present a medical problem, clinicians are often faced with a challenge of achieving gingival esthetics.

**Materials and Methods:**

A method of de-epithelialization of the pigmented gingiva using Kirkland’s gingivectomy knife is described. Twenty patients who were conscious about their gingival melanin pigmentation were selected. The gingiva of the whole of the arch was abraded until the entire visible pigmentation was removed. Clinical observations for intensity of pigmentation were recorded at baseline and then after surgery at monthly intervals over a period of 9 months according to Dummett-Gupta Oral Pigmentation Index scoring criteria proposed by Dummett C. O. in 1964.

**Results:**

The mean gingival melanin pigmentation score came down to 0.407 after 9 months as compared to preoperative score, which was 2.24. No repigmentation occurred in fair-complexioned persons. In persons with wheatish complexion, repigmentation was seen in 85.71% of the cases, but scores came down to 0.38 postoperatively as compared to 2.27 preoperatively. In dark-complexioned persons, repigmentation occurred in all cases, but the mean scores were 0.93 as compared to 2.40 preoperatively. The difference between preoperative and postoperative mean scores for each segment was put to statistical analysis by applying paired *t* test and was found to be significant.

**Conclusion:**

As this method has shown statistically significant results, it can be used in patients who are conscious of pigmented gingiva and want an esthetically satisfactory color.

## INTRODUCTION

Esthetics has become an important aspect of dentistry, and clinicians have to face the challenge of achieving acceptable gingival esthetics, along with addressing biological and functional problems. The color of the gingiva plays an important role in overall esthetics. As seen clinically, it varies from person to person, in different areas of the mouth and appears to be correlated with the color of the skin. According to Dummett (1959),[1] color of the healthy gingiva is variable, ranging from pale pink to deep bluish purple. Between these limits of normalcy are a large number of colors, which depend primarily upon the depth of epithelialization, the degree of cornification, arrangement of vascularity and the degree of melanogenesis.

The gingivae are the most frequently pigmented of the intraoral tissues, in addition to being the most readily seen during inspections. The most frequent cause of gingival pigmentation is melanin, though other pigments, such as carotene, oxyhemoglobin and reduced hemoglobin, which contribute to the normal color of the integument, are also found in the masticatory mucosa.[2] Melanin is the fundamental pigment that colors the tissues. It appears as early as 3 hours after birth in the oral tissues and in some cases is the only sign of pigmentation on the body.[3] It is a non–hemoglobin-derived pigment formed by the cells called melanocytes, which are dendritic cells of neuroectodermal origin located in the basal and spinous layers of the gingival epithelium. Melanin granules are phagocytosed and contained within other cells of the epithelium and connective tissue, called melanophages or melanophores.[4] It is generally accepted that pigmented areas are present only when melanin granules synthesized by melanocytes are transferred to keratinocytes. This close relationship between melanocytes and keratinocytes was labeled by Fitzpatrick and Breathnach as the epidermal-melanin unit.[5]

Gingival hyperpigmentation is seen as a genetic trait in some populations and is more appropriately termed physiologic or racial gingival pigmentation.[6] It has also been suggested that although pigmentation under normal conditions is genetically determined, its particular distribution in the mouth may be the result of secondary influences and perhaps environmental factors. High levels of oral melanin pigmentation are normally observed in individuals of African, East- Asian or Hispanic ethnicity.[7] Although more prevalent among blacks, oral melanosis has been demonstrated in other races also. In general, individuals with fair skin will not demonstrate overt tissue pigmentation, although comparable numbers of melanocytes are present within their gingival epithelium. The melanocytes are generally inactive or hypoactive in melanin synthesis.[5]

Although this pigmentation of the gingiva is physiologic and does not present any medical problem, people at large do not appreciate dark-colored gingiva, and demand is made for the removal of broad black zone of pigmentation from gums for esthetic reasons. Ginwalla *et al*. (1966)[8] described the broad black zone of pigmentation on the gingiva as “unsightly” and suggested its removal. A questionnaire survey by Dummett (1969)[9] to explore personal attitude towards gingival pigmentation showed that “pink gum” is the ideal one.

Various procedures have been attempted in the past to remove gingival melanin pigmentation, such as by chemicals,[10] abrasion with diamond bur,[11,12] gingivectomy,[5,13,14] soft tissue autograft,[7] partial-thickness flap,[15] cryosurgery[16] and lasers.[17,18] These techniques have shown variable results, and each technique has its own advantages and inadequacies. Dummett and Bolden (1963)[14] operated upon pigmented gingiva by gingivectomy in 9 cases. Repigmentation occurred in 66.66% of the cases between 33 to 120 days. Ginwalla *et al*. (1966)[8] used 3 procedures for depigmentation — slicing, bone denudation and abrasion. Sameer (2006)[12] treated 3 cases of gingival hyperpigmentation by abrasion with a high-speed hand piece and diamond bur and reported no repigmentation in an 18-month follow-up period; but in a similar technique, Farnoosh reported slight repigmentation in 2 cases.[11] A free gingival graft can also be used to eliminate pigmented areas, but it is an extensive procedure which requires an additional surgical site (donor site) and color matching. Tal *et al*. (1987)[16] used cryosurgery for the removal of gingival pigmentation and reported no repigmentation up to a 20-month follow-up period. Atsawasuwan *et al*. (2000)[17] used Nd:YAG laser for the treatment of hyperpigmented gingiva and reported no recurrence in a period of 11 to 13 months of follow-up, but suggested that Nd:YAG laser should be used cautiously. The laser and cryosurgical treatment modalities achieved satisfactory results, but they require sophisticated equipment, which is not commonly available in hospitals and clinics, and also they are expensive for the patient. So these techniques are not widely accepted or popularly used.

One of the earlier and still popular techniques is the surgical removal of the undesirable pigmentation using scalpel. The surgical depigmentation procedure essentially involves the removal of gingival epithelium along with a layer of the underlying connective tissue and allowing the denuded connective tissue to heal by secondary intention. The new epithelium that forms is devoid of melanin pigmentation.[19] But removal of gingival melanin pigmentation should be performed cautiously, and the adjacent teeth should be protected since inappropriate technique may cause gingival recession and damage to underlying periosteum and bone.

In the present study, an effort has been made to assess the procedure to free the gingiva of melanin pigmentation by deepithelialization with Kirkland’s gingivectomy knife and to note the duration and intensity of repigmentation. The method used is comparatively simple, safe and nonaggressive and can be easily repeated if necessary.

## MATERIALS AND METHODS

**Figure 1:**
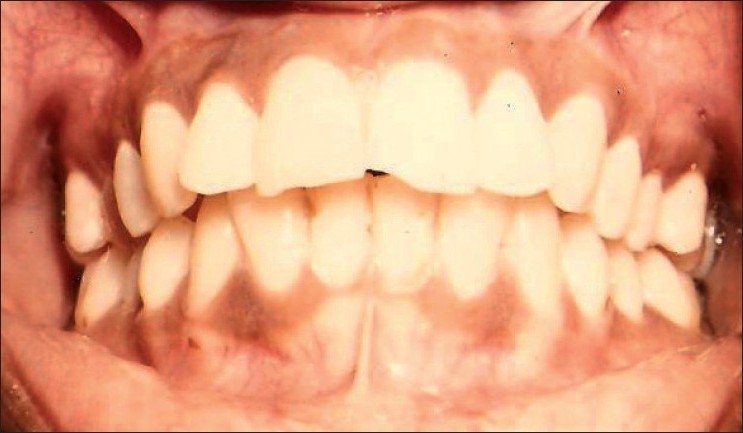
Preoperative photograph showing gingival melanin hyperpigmentation

Twenty patients in the age group of 17-30 years who were conscious of their gingival melanin pigmentation and wanted its removal were selected [Figure 1]. A complete medical history and investigations for blood and urine were carried out to rule out any systemic contraindications for the surgery. The procedure was carried out in the maxillary arch as it is esthetically more important. Arch was divided into 3 segments (segment I- right posterior segment, segment II- anterior segment and segment III- left posterior segment). Patients were placed in 3 groups according to their facial complexion, viz., fair, wheatish or brown and dark. Out of 20 patients, 3 were fair; 14, wheatish; and 3, dark complexioned. Preoperative and postoperative observations about the gingival melanin pigmentation were made according to Dummett-Gupta Oral Pigmentation Index scoring criteria given by Dummett C.O. in 1964[20]:

0 — no clinical pigmentation (pink gingiva)
1 — mild clinical pigmentation (mild light brown color)
2 — moderate clinical pigmentation (medium brown or mixed pink and brown color)
3 — heavy clinical pigmentation (deep brown or bluish black color)

Score in each tooth was taken including one full interdental papilla. Observations were made in natural light. The assessment of complexion and the scoring of the pigmentation index were done by all the three examiners, and the agreedupon score was assigned to prevent individual variations.

**Figure 2:**
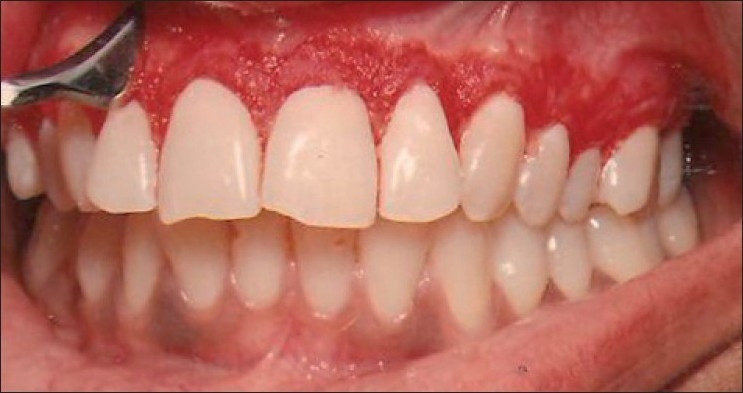
Photograph showing gingival depigmentation using Kirkland’s knife

**Figure 3:**
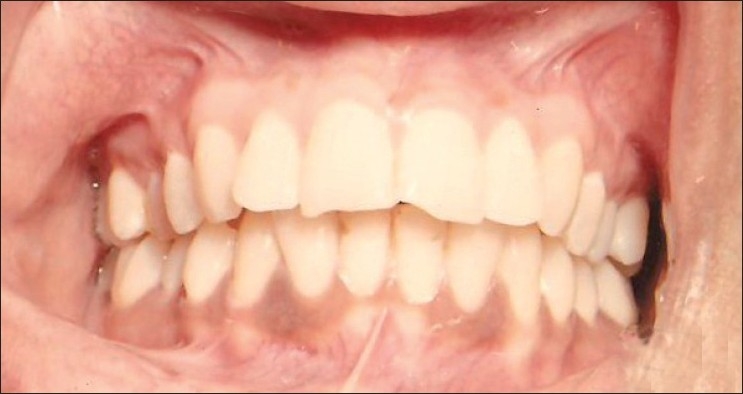
Postoperative photograph 9 months after gingival depigmentation

Under perfectly aseptic conditions and infiltration anesthesia, the gingiva of the facial surface of the whole of the maxillary arch was abraded with Kirkland’s gingivectomy knife starting from the distal surface of the last tooth on the right side to the distal surface of the last tooth on the left side. The Kirkland’s knife was used [Figure 2] for scraping of the gingiva because it is more convenient to use and also the accessibility in the posterior areas of the arch is easy as compared to scalpel blade or hand piece with diamond bur. The entire visible pigmentation was removed, exposing the underlying connective tissue. After that the raw connective tissue surface was examined by a magnifying mouth mirror and magnifying lens to locate any visible remaining pigmentation for its complete removal, as much as possible. In areas where gingiva was thin, scraping was done carefully to avoid exposure of the bone. Bleeding was controlled by pressure with sterile gauze. Then, the area was covered with periodontal dressing for 7 days and postoperative instructions were given. Patients were recalled at 7, 15 and 21 days postoperatively to observe healing. Observations for clinical reappearance of gingival melanin pigmentation and its intensity were recorded on completion of the first month, and after that at monthly intervals over a period of 9 months postoperatively [Figure 3].

### Statistical analysis

Statistical analysis was carried out using Statistical Package for Social Sciences (SPSS Inc., Chicago, IL, version 11.0 for Windows). For every quantitative variable, mean and standard deviation were calculated. Means were compared of two groups using the Student *t* test. For time-related variables, paired *t* test was applied. Qualitative or categorical variables were described as frequencies and proportions. Statistical tests were two sided and performed at a significance level of α=.05.

## RESULTS

Out of 20 patients, repigmentation appeared in 15 patients over a 9-month observation period after surgery [Table 1]. Repigmentation here does not mean that the whole of the segment or arch was pigmented, but even a small dot or streak in relation to a single tooth was considered as repigmentation in that segment and even in that individual case. It appeared at different times in each patient and was of varying intensity, in the form of very small spots, dots and streaks of mild intensity as compared to broad heavy bands seen preoperatively.

**Table 1:** Percentage of cases showing gingival melanin repigmentation and preoperative and postoperative mean scores of gingival melanin pigmentation at different observation times

No. of cases operated	Duration of observation after	Repigmentation after surgery				Gingival melanin pigmentation mean score	
		Cases with repigmentation		Cases with no repigmentation		Pre-operatively	Post-operatively
		No.	% age	No.	% age		
20	1 month	1	5	19	95	2.24	0.003
	2 months	1	5	19	95		0.01
	3 months	6	30	14	70		0.08
	4 months	11	55	9	45		0.154
	5 months	12	60	8	40		0.19
	6 months	12	60	8	40		0.235
	7 months	13	65	7	35		0.302
	8 months	15	75	5	25		0.375
	9 months	15	75	5	25		0.407

The preoperative gingival melanin pigmentation mean scores for segments I, II and III were 2.01, 2.69 and 2.02, respectively; and for the whole arch, the score was 2.24. Postoperatively after 9 months, the gingival melanin pigmentation mean scores of segments I, II and III reduced to 0.44, 0.45 and 0.33, respectively; and for the whole arch, the score reduced to 0.407 [Tables 2 and 3]. The difference between preoperative and postoperative gingival melanin pigmentation mean scores was put to statistical analysis by applying *t* test and was found to be statistically significant [Table 4].

**Table 2:** Pre-operative gingival melanin pigmentation mean scores

Case no.	Segment 1	Segment 2	Segment 3	Full arch
1	2.60	3.00	2.60	2.73
2	2.00	3.00	2.00	2.33
3	1.60	2.66	1.60	1.95
4	2.50	3.00	2.40	2.63
5	1.50	3.00	1.50	2.00
6	2.50	3.00	2.75	2.75
7	2.80	3.00	2.20	2.66
8	2.25	2.83	2.50	2.52
9	1.80	3.00	2.00	2.26
10	2.60	2.66	2.60	2.62
11	2.60	3.00	2.60	2.73
12	2.80	3.00	2.40	2.73
13	2.60	3.00	2.60	2.73
14	2.00	1.83	1.80	1.87
15	1.20	3.00	1.25	1.82
16	2.00	2.83	1.80	2.21
17	1.60	2.83	1.80	2.07
18	1.75	2.00	1.50	1.75
19	0.75	1.33	1.00	1.02
20	0.75	2.00	1.50	1.41
Mean	2.01	2.69	2.02	2.24

**Table 3:** Post-operative gingival melanin pigmentation mean scores after 9 months

Case no.	Segment 1	Segment 2	Segment 3	Full arch
1	1.40	1.50	1.20	1.36
2	0.00	0.00	0.00	0.00
3	0.40	0.16	0.20	0.25
4	0.75	0.83	0.60	0.72
5	0.00	0.00	0.00	0.00
6	0.25	0.16	0.25	0.22
7	0.60	0.83	1.00	0.81
8	0.00	0.00	0.25	0.08
9	0.00	0.16	0.00	0.05
10	1.40	0.83	0.40	0.87
11	0.80	1.00	0.40	0.73
12	0.40	0.83	0.40	0.54
13	0.00	0.00	0.00	0.00
14	1.00	0.50	0.60	0.70
15	0.60	0.83	0.50	0.64
16	0.40	0.66	0.40	0.48
17	0.60	0.33	0.40	0.44
18	0.25	0.50	0.00	0.25
19	0.00	0.00	0.00	0.00
20	0.00	0.00	0.00	0.00
Mean	0.44	0.45	0.33	0.407

**Table 4:** Statistical significance of difference between pre and post-operative gingival melanin pigmentation mean scores

Area	Gingival melanin pigmentation score		Difference between the means	% age of difference between the mean scores	Value of ‘t’	Whether significant	Level of significance
	Pre-operative	Post-operative					
Segment 1	2.01	0.44	1.57	128.68	11.69	Yes	0.01
Segment 2	2.69	0.45	2.24	142.67	17.77	Yes	0.01
Segment 3	2.02	0.33	1.69	144.44	14.95	Yes	0.01
Arch full	2.24	0.407	1.833	138.63	13.09	Yes	0.01

0.01 — statistically significant at 1% level of reliability.

As the focus was on the anterior segment, the results were quite encouraging. Preoperatively, 13 patients were having heavy pigmentation and 7 were having moderate pigmentation in the anterior segment, but postoperatively no patient showed heavy repigmentation. Seven patients showed no repigmentation at all; and in 9 patients small spots of mild intensity and in 4 patients moderate repigmentation in the form of small spots or streaks were observed [Table 5].

**Table 5:** Pre and post-operative gingival melanin pigmentation in the anterior region

Intensity of pigmentation	Gingival melanin pigmentation			
	Pre-operative		Post-operative	
	No. of cases	% age	No. of cases	% age
Heavy	13	65	Nil	Nil
Moderate	7	35	4	20
Wild	Nil	Nil	9	45
Mo pigmentation	Nil	Nil	7	35

All (100%) the 3 patients with dark complexion had repigmentation, whereas 12 (85.71%) out of 14 wheatish-or brown-complexioned patients had repigmentation after surgery. None of the 3 fair-complexioned patients had repigmentation. Postoperatively after 9 months, gingival melanin pigmentation score decreased from 2.40 to 0.93 in dark-complexioned patients, from 2.27 to 0.38 in wheatish- or brown-complexioned patients and from 1.91 to 0.000 in faircomplexioned patients [Table 6].

**Table 6:** Gingival melanin repigmentation after 9 months post-operatively in relation to facial complexion

Complexion	No. of cases	Repigmentation		Gingival melanin pigmentation mean score	
		No. of cases	% age	Preoperative	Postoperative
Fair	3	0	0.00	1.91	0.00
Wheatish or brown	14	12	85.71	2.27	0.38
Dark	3	3	100	2.40	0.93

## DISCUSSION

Gingival hyperpigmentation is a condition of major concern for a large number of patients, and periodontists are often confronted with the problem of negative impacts of dark gingivae. Several treatment modalities have been suggested in the literature, with varying results, but the repigmentation has been documented to occur. The large variation in time of repigmentation may be related to the technique used and the race of the patient. The repigmentation is described as spontaneous. The mechanism of repigmentation is not understood; but according to the migration theory, active melanocytes from the adjacent pigmented tissues migrate to the treated areas, causing repigmentation.[13] An attempt has been made in the present study to minimize the migration of melanocytes from adjacent gingival margins of the operated area. So instead of scraping the epithelium from the gingiva of anterior segment only, the gingiva of the whole of the arch was abraded. Another reason for repigmentation may be the melanocytes which are left during surgery. These may have become activated and started synthesizing melanin. Ginwalla *et al*. (1966)[8] also attributed the repigmentation to left-out melanocytes.

Results of this study showed no repigmentation in 25% of the cases until 9 months postoperatively. Though repigmentation was observed in 75% of the cases at varying time intervals, the gingival pigmentation mean score reduced to 0.407 postoperatively after 9 months from 2.24 preoperatively. The repigmentation appeared in different areas in different individuals at varying times during the observation period. Ginwalla *et al*. reported repigmentation in 50% of their cases between 24 and 55 days.[8] Perlmutter and Tal have also reported gingival repigmentation, which occurred 7 years after removal of gingival tissues in 1 patient.[13] All the patients were explained about the possibility of repigmentation, so all except one were satisfied with the results as the repigmentation, wherever appeared, was of very mild intensity in the form of very small spots, dots or streaks without creating any esthetic problem, as compared to dark continuous bands of heavy or moderate intensity preoperatively. In the anterior segment, the melanin pigmentation mean score came down to 0.45 from 2.69 preoperatively. Though these results were satisfying, it was also observed that repigmentation in the anterior segment was more as compared to the posterior segments. This difference may be attributed to the effect of sunlight on the anterior segment. Raut *et al*. (1954)[21] also showed that anterior attached gingiva, which was more exposed to light, was more pigmented than the posterior one.

In the present study, repigmentation was also compared in relation to facial complexion. The preoperative gingival melanin pigmentation mean scores in the fair-complexioned patients were less (1.91), as compared to wheatish- or brownand dark- complexioned patients, in whom the mean score was 2.27 and 2.40, respectively. Postoperatively, it was concluded that people with fair complexion have lesser chances of developing repigmentation; as in the present study, none (0.00%) of the 3 fair-complexioned patients developed repigmentation, whereas all (100%) of the 3 dark-complexioned patients had repigmentation. Out of 14 patients with wheatish or brown complexion, repigmentation was observed in 12 (85.71%) patients. Gingival melanin repigmentation mean score was also higher in the dark-complexioned patients (0.93) as compared to wheatish- or brown-complexioned patients (0.38). The color of gingiva has been correlated with facial complexion. Raut *et al*. (1954)[21] showed that the degree and the incidence of pigmentation of the gingiva increase as the complexion changes to the darker shade. This may be applied to the above findings even for repigmentation in cases with different facial complexions, and the possible reason may be the rate of melanogenesis which is intrinsically maintained and is higher in dark-complexioned patients as compared to lightcomplexioned patients.[22] This relationship of duration and intensity of repigmentation with the complexion of a person must be taken into consideration while comparing the results of various studies done in different races.

## CONCLUSION

From the above discussion, it is concluded that although repigmentation appeared in some cases in different areas at different times, yet due to its mild intensity, the results can be considered to be satisfying, which is the ultimate goal of therapy. Further it is thought that even if repigmentation occurs, it will take some time to become dark enough to make the individual conscious again; hence this procedure is quite valuable for persons who are conscious about their appearance and personality, especially females, more so at their matrimonial age. The procedure adopted is also very simple, cost effective and less painful, and the amount of tissue removed is very less; also, the procedure can be repeated without any complication if the pigmentation in any area re-occurs. But further research with longer periods of clinical observations, along with histological studies to observe the cause and the factors affecting the rate of repigmentation, is needed.
